# Increased Inter-Colony Fusion Rates Are Associated with Reduced COI Haplotype Diversity in an Invasive Colonial Ascidian *Didemnum vexillum*

**DOI:** 10.1371/journal.pone.0030473

**Published:** 2012-01-31

**Authors:** Kirsty F. Smith, Lauren Stefaniak, Yasunori Saito, Chrissen E. C. Gemmill, S. Craig Cary, Andrew E. Fidler

**Affiliations:** 1 Cawthron Institute, Nelson, New Zealand; 2 Department of Biological Sciences, University of Waikato, Hamilton, New Zealand; 3 Department of Marine Sciences, University of Connecticut, Groton, Connecticut, United States of America; 4 Shimoda Marine Research Center, University of Tsukuba, Shimoda, Shizuoka, Japan; University of Canterbury, New Zealand

## Abstract

Considerable progress in our understanding of the population genetic changes associated with biological invasions has been made over the past decade. Using selectively neutral loci, it has been established that reductions in genetic diversity, reflecting founder effects, have occurred during the establishment of some invasive populations. However, some colonial organisms may actually gain an ecological advantage from reduced genetic diversity because of the associated reduction in inter-colony conflict. Here we report population genetic analyses, along with colony fusion experiments, for a highly invasive colonial ascidian, *Didemnum vexillum*. Analyses based on mitochondrial cytochrome oxidase I (COI) partial coding sequences revealed two distinct *D. vexillum* clades. One COI clade appears to be restricted to the probable native region (i.e., north-west Pacific Ocean), while the other clade is present in widely dispersed temperate coastal waters around the world. This clade structure was supported by 18S ribosomal DNA (rDNA) sequence data, which revealed a one base-pair difference between the two clades. Recently established populations of *D. vexillum* in New Zealand displayed greatly reduced COI genetic diversity when compared with *D. vexillum* in Japan. In association with this reduction in genetic diversity was a significantly higher inter-colony fusion rate between randomly paired New Zealand *D. vexillum* colonies (80%, standard deviation ±18%) when compared with colonies found in Japan (27%, standard deviation ±15%). The results of this study add to growing evidence that for colonial organisms reductions in population level genetic diversity may alter colony interaction dynamics and enhance the invasive potential of newly colonizing species.

## Introduction

Human mediated transfer of species into regions beyond their native range, and the resulting ecosystem perturbations, are major contributors to currently accelerating rates of indigenous biodiversity loss and species extinction [1]–[3]. In recent years, considerable progress has been made in describing terrestrial and aquatic bioinvasions in terms of the physical mechanisms of propagule transport, phylogenetic relationships, and population genetics [4]–[6]. Population genetic theory predicts that the small founder populations typical of invasive species, at least in the initial colonization stages, contain an unrepresentative portion of the total genetic variation present in the source population [7], [8] and that further stochastic losses of allelic diversity (i.e. genetic drift) are anticipated in such small populations [4], [7], [9].

Such non-adaptive changes in the genetics of newly colonizing species are expected to have both genotypic and phenotypic consequences, possibly including deleterious inbreeding depression [10], [11]. In addition, a colonizing species is exposed to novel selection pressures in its new environment with its extant genetic diversity strongly influencing its capacity to adaptively evolve in response to these selective pressures [4], [10], [12]. Despite these theoretical predictions, many introduced species with associated genetic bottlenecks have successfully established in new environments and have often out-competed locally-adapted native species [13]–[15]. Furthermore, reduced genetic diversity in invasive populations is not as common as initially expected; with a recent review concluding only 37% of studies on aquatic invasions reported evidence of significant loss of genetic diversity in the introduced populations [9]. In some cases invasive populations actually appear to have increased genetic diversity in their new environment which has been attributed to admixture of lineages from multiple native populations with differing genetic profiles [16]–[18].

Biological explanations for the invasive success of particular species can be elusive and general ecological parameters such as release from predation [19], reduced competition [20], or naïve prey in the new environment [21] are often cited as primary causes of invasion success. More recently, progress has been made in identifying specific phenotypic traits that may confer increased invasion success for particular taxa [22]–[26]. A useful approach to identifying phenotypic traits relevant to invasion success is to compare and contrast biological characteristics of a species in both native and non-native regions [27]. Taking this strategy, in this study we compared both the population genetics and the colony fusion biology of a highly invasive colonial ascidian, *Didemnum vexillum*, within both its probable native region (Japan) and a recently established, non-native region (New Zealand).

Genetic loci that strongly influence the structure and functioning of biological colonies have been characterized to the molecular level from a diverse range of phyla: Arthropoda [28], Mycetozoa [29], and Chordata [30]. In these examples, a small number of highly polymorphic loci have been described that have profound effects on inter-colony interactions [28]–[30]. In the context of invasion biology, a potential consequence of such polymorphic genetically based recognition systems is that reduced genetic diversity may actually enhance invasiveness, at least in the short-term, by reducing intra-specific conflict and increasing mean colony size. A well-documented example of such a mechanism is the highly invasive Argentine ant (*Linepithema humile*) which displays both reduced genetic diversity and reduced inter-colony aggression in non-native regions [26], [31], [32]. In newly introduced populations of Argentine ant founder effects reduce both genetic diversity and intra-specific aggression, enabling the formation of the very large colonies typical of this invasive species [26], [33].

Taxonomically diverse groups of colonial ascidians have been shown to have allorecognition mechanisms that distinguish closely related colonies from unrelated colonies [34]–[39]. Adjacent, genetically similar colonies are able to fuse to form a single chimeric colony, while genetically dissimilar colonies will not fuse. For the well-studied colonial botryllid ascidian, *Botryllus schlosseri*, fusion to produce a chimeric colony is a well-described and complex process involving both the tunic and the vascular system that connects the individual zooids that comprise the colony [34]–[39]. In contrast, the colony fusion process of didemnid ascidians, which lack a common vascular system, is poorly understood [40]. Whatever the details of the fusion process, the resulting chimeric colonies are expected to possess greater genetic variability and possibly display an associated wider range of physiological tolerances [41]. In *B. schlosseri* a single highly polymorphic locus has been shown to strongly influence colony allorecognition [39], [42], [43]. In such systems, significant reductions in allelic diversity, specifically at allorecognition locus/loci, may have a profound effect on the probability of two randomly-selected colonies in a population being able to fuse, which in turn may influence typical colony sizes and structure [39], [44]. This hypothesis has been previously proposed to explain why some colonial ascidians are such successful invaders [45]. Colonial ascidians appear to be a promising taxonomic group for investigating mechanisms that link population genetics to phenotypic differences in invasive populations.

Over the past decade, morphologically similar colonies of an ascidian, *Didemnum* sp., from diverse geographical temperate regions have been reported: New Zealand [46], [47], European Atlantic coast [48]–[50] and the west and east coasts of North America [51]. These various populations were initially designated as five different, and previously described, *Didemnum* species along with two entirely new species designations for New Zealand (*D. vexillum* Kott, 2002) and New Hampshire (*D. vestum* Kott, 2004) [49]. However, subsequent morphological [49] and molecular comparisons [52] concluded that all specimens belonged to a single species: *Didemnum vexillum* Kott, 2002 [49], [52]. These authors suggested that the native range of *D. vexillum* was likely to be in the northwest Pacific [49], [52]. In New Zealand, and some other regions in which *D. vexillum* is considered non-native (e.g. the east coast of North America), *D. vexillum* often forms very large colonies that may smother other marine invertebrates including commercially valuable aquaculture species [53]. For example, on Georges Bank, east of Cape Cod, Massachusetts, *D. vexillum* colonies now extend over very large areas - in 2005 estimated as >230 km^2^ [54].

*Didemnum vexillum* was first reported in New Zealand in 2001 within two adjacent harbours on the North Island, Tauranga and Whangamata [46]. Later that same year, *D. vexillum* was identified in the Marlborough Sounds (South Island), initially on a single barge that had earlier been moved from Tauranga harbour [55]. Current evidence strongly suggests that *D. vexillum* is not native to New Zealand including; a well-defined history of spread, the strong correlation between the distribution of *D. vexillum* in New Zealand and the infested barge, and that *D. vexillum* arguably meets all ten of the accepted criteria for designating a species as non-native [53], [56]. Given the tendency of *D. vexillum* in New Zealand to form large, biofouling colonies [53], we hypothesized that the colonization of New Zealand by *D. vexillum* was associated with a significant reduction in its genetic diversity and in consequence New Zealand populations may exhibit increased rates of inter-colony fusion. To test this hypothesis, we examined genetic diversity within the *D. vexillum* mitochondrial cytochrome oxidase I (COI) coding region, comparing samples collected from New Zealand with those from Japan. In parallel we assessed the frequencies with which randomly selected *D. vexillum* colonies fused in experimental cut surface assays, comparing populations in New Zealand and Japan.

## Results

### *Didemnum vexillum* COI haplotypes in New Zealand and Japan

**Figure 1:**
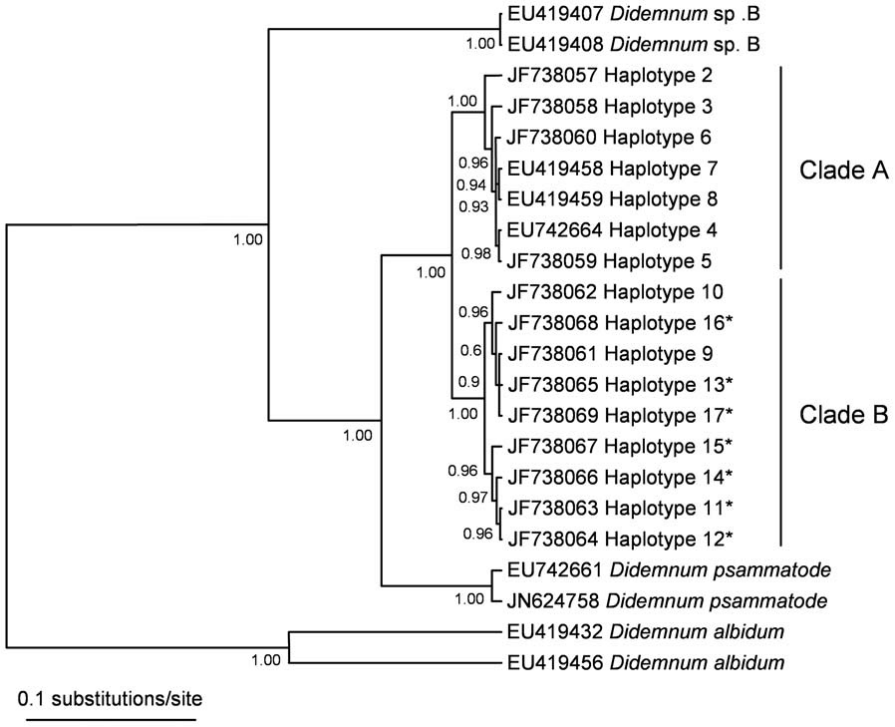
Bayesian phylogenetic analysis of 16 *Didemnum vexillum* cytochrome oxidase I (COI) haplotypes. Numbers on nodes denote posterior probability values. GenBank accession numbers of the COI haplotypes are shown. Asterisks indicate seven COI haplotypes (H11–H17) not previously reported by Stefaniak et al. (2009) [52]. COI sequences from *Didemnum psammatode* (EU742661, JN624758), *D. albidum* (EU419432, EU419456) and *Didemnum* sp. B (EU419407, EU419408) were used as outgroups.

Tissue sample from 98 colonies morphologically identified as being *D. vexillum* were obtained: Japan, four locations, n = 37 colonies; New Zealand, five locations, n = 61 colonies (Figure S1, Table S1). Mitochondrial COI partial coding sequences (586 bp) were amplified from all 98 specimens. When combined with the previously reported *D. vexillum* COI sequence dataset [52] (n = 71) the resulting 169 COI sequences grouped into 16 haplotypes, denoted H2–H17. Of the 13 COI haplotypes observed in this study (haplotypes H2, H3, H5, H6, H9–H17; corresponding source locations and GenBank accession numbers listed in the supplementary materials Table S1), seven (haplotypes H11–H17) were not previously reported [52] (Figure 1). All sequence differences between the 13 COI haplotypes were synonymous.

### Phylogenetic analyses of *Didemnum vexillum* COI haplotypes

**Figure 2:**
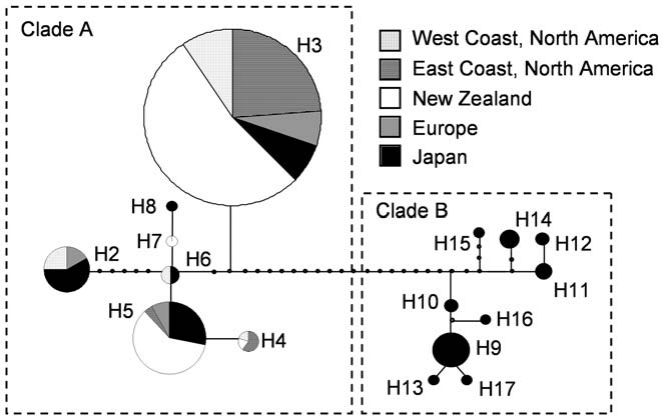
Statistical parsimony network of 16 *Didemnum vexillum* cytochrome oxidase I (COI) haplotypes. Areas of circles are proportional to the frequency of each haplotype in the dataset and differing shading indicates five different geographic regions as indicated. Small black circles on the branches indicate hypothetical intermediate haplotypes that were not observed.

Bayesian phylogenetic analysis of the 16 *D. vexillum* COI haplotypes revealed two well supported distinct clades, denoted A and B, each supported by posterior probability values of 1.0 (Figure 1). The clear separation of these two COI clades was also apparent in a statistical parsimony network (Figure 2) with 17 hypothetical mutational steps separating the basal nodes of the two clades (Figure 2). Clade A (haplotypes H2–H8) included sequences from five broad sampling regions: New Zealand (NZ), Japan, West Coast North America (WCNA), East Coast North America (ECNA) and Europe (Figure 2). In contrast, the Clade B haplotypes (i.e. H9–H17) were exclusively amplified from the Japanese samples (Table S1, Figure 2).

The percentages of polymorphic sites within clades A and B were 1.9% and 2.6%, respectively, while the percentage polymorphic sites within the combined COI sequence dataset was 6.8%. Divergence between clades A and B, as estimated by *p*-distance, is 0.040 while the number of net nucleotide substitutions per site (*D*a) between clades A and B is 0.032.

To investigate if the *D. vexillum* COI sequence based clade structure was reflected in the nuclear genome, 18S rDNA sequences were amplified from DNA preparations corresponding to the thirteen COI haplotypes found in this study (i.e. haplotypes H2, H3, H5, H6, H9–H17). All 18S ribosomal DNA (rDNA) sequences amplified were identical except at position 705; the 18S rDNA sequences amplified from clade A genomic DNA (COI haplotypes H2, H3, H5 and H6) were A_705_ while the clade B haplotypes (i.e. H9–H17) were G_705_ (coordinates of GenBank accession number JF738070).

### Population genetic analyses

For population genetic analyses, the *D. vexillum* COI sequences from both this work and Stefaniak et al. (2009) [52] (n = 169 in total) were grouped into five distinct geographic regions: NZ (n = 67), Japan (n = 50), WCNA (n = 15), ECNA (n = 27) and Europe (n = 10) (Table 1). To allow for the possibility that the COI sequences of clades A and clade B are derived from two cryptic sibling species, population genetic analyses were carried out using two datasets: the first consisted exclusively of clade A COI sequences (i.e. n = 144 sequences, haplotypes H2–H8) while the second dataset consisted of both the clade A and B sequences combined (i.e. n = 169 sequences, haplotypes H2–H17) (Table 1).

**Table 1:** *Didemnum vexillum* cytochrome oxidase I (COI) sequence diversity measures from five geographic regions.

Population	First record*	No. of colonies	No. of haplotypes	No. of private haplotypes	Haplotype richness	Nucleotide diversity (± std. dev.)	Haplotype diversity (± std. dev.)
NZ	2001 (Whangamata)	67	3	0	2.3	0.003 (±0.002)	0.376 (±0.058)
Japan (A+B)	1926 (Mutsu Bay)	50	15	11	9.4	0.024 (±0.012)	0.902 (±0.019)
Japan (A)	1926 (Mutsu Bay)	25	6	2	6.0	0.007 (±0.004)	0.787 (±0.040)
WCNA	1993 (San Francisco)	15	4	0	3.9	0.006 (±0.004)	0.619 (±0.120)
ECNA	1982 (Damariscotta R.)	27	3	0	2.6	0.002 (±0.002)	0.271 (±0.105)
Europe	1998 (the Netherlands)	10	3	0	3.0	0.007 (±0.004)	0.622 (±0.138)

Sequence dataset is combined from this study and Stefaniak et al. (2009) [52].

*Dates from Lambert (2009) [49]. Abbreviations: Japan (A+B), Japan Clade A and B data; Japan (A), Japan Clade A data only; NZ, New Zealand; WCNA, West Coast North America; ECNA, East Coast North America.

H3 was the only COI haplotype found in all five geographical regions and appears to be the most common world wide (Figure 2). In this study, only two clade A COI haplotypes, H3 and H5, were found within the 61 NZ samples examined (Figure 2, Table 1). Despite extensive sampling from the Marlborough Sounds, NZ (n = 44), we did not recover the H4 haplotype that had previously been reported from the barge that is believed to have transported *D. vexillum* to the Marlborough Sounds [52]. The paucity of COI haplotypes in NZ (n = 3 haplotypes) is reflected in lower haplotype and nucleotide diversity values when compared with the four other geographical regions (Table 1). ECNA, Europe, and WCNA follow NZ in having low levels of COI haplotype diversity when compared with Japan (Table 1). Of the fifteen COI haplotypes found in Japan, eleven are apparent private haplotypes (H7, H8, H9–H17) with nine of these private haplotypes (H9–H17) placed in clade B (Figure 2, Table 1). No other geographic region had private haplotypes (Table 1). When both clade A and B haplotypes were included in the analysis, Japan had the highest levels of haplotype richness, nucleotide and haplotype diversity (Table 1). In contrast, when the data-set was restricted to the clade A haplotypes, Japan had similar haplotype and nucleotide diversity to WCNA and Europe but haplotype richness was still much higher in Japan (Table 1). ECNA resembles NZ in having very low levels of COI haplotype richness and nucleotide and haplotype diversity (Table 1).

### Cut surface assays (CSA) to determine *D. vexillum* inter-colony fusion compatibility

Four independent CSA experiments were conducted in New Zealand between February and April 2009 (i.e. austral summer/autumn) with an average of eleven CSA pairings (range 21–3), excluding positive controls, for each experiment (Table S2A, Table 2). Positive controls consisted of autogenic parings of samples from the same colony and all positive controls resulted in fusions (Table S2). In New Zealand an average of 80% (standard deviation ±18, range 60–100%) of the CSA pairings resulted in inter-colony fusions (Table 2). Three independent CSA experiments were conducted in Japan in July 2010 with ten pairings for each experiment (Table S2B, Table 2) and an average inter-colony CSA fusion rate of 27% (standard deviation ±15%, range 10–40%, Table 2). CSA determined fusion rates in the New Zealand experiments were significantly higher than in Japan (z = 4.53, *P* = 0.00). There was no significant difference between fusion rates for any experiments within either NZ or Japan. Amplified COI sequences established that all the colonies used in the CSA fusion experiments were from *D. vexillum* clade A (NZ: haplotypes H3 or H5; Japan: haplotypes H2 or H5; Table S2).

**Table 2:** Summary of results of cut surface assays (CSA) of *Didemnum vexillum* carried out in New Zealand and Japan.

Location	Number of CSA*	Number of fusions	Percentage fused
**New Zealand**			
27 Feb 2009	10	7	70
06 Mar 2009	21	19	90
25 Mar 2009	10	6	60
06 Apr 2009	3	3	100
Average	11	8.75	80
Standard deviation			18
**Japan**			
09 Jul 2010	10	4	40
12 Jul 2010	10	1	10
13 Jul 2010	10	3	30
Average	10	2.67	27
Standard deviation			15

*Excludes autogenic (positive control) pairings.

## Discussion

*Didemnum vexillum* COI haplotype diversity within New Zealand is significantly less than within *D. vexillum*'s putative native region in Japan. The New Zealand *D. vexillum* populations sampled contained as few as three COI haplotypes. These data suggest an interpretation that *D. vexillum* experienced a significant reduction in genetic diversity when colonizing New Zealand, presumably due to a founder effect. Other putatively introduced populations (i.e., Europe, ECNA, WCNA) also display reduced diversity when compared to Japan, although sample sizes from these regions are small. Reduced genetic diversity is a common, but by no means universal, feature of invasive populations [9]. The genetic diversity of non-native *D. vexillum* populations will increase with time as further colonization events augment existing genetic variation, although this may not be always be the case [57]. Mitochondrial sequences, being haploid and almost exclusively maternally inherited, are more sensitive to founder events than bi-parentally inherited, diploid nuclear genes [58]. However, the reductions in COI genetic diversity detected in the New Zealand *D. vexillum* clade A population are potentially associated with reductions in allelic diversity at nuclear loci [59].

Phylogenetic analyses of the sixteen *D. vexillum* COI haplotypes revealed a well-supported monophyletic *D. vexillum* species grouping, composed of two distinct well-supported clades, termed A and B. Clade A included haplotypes from all five widely-separated geographical regions defined in this study while clade B was composed of haplotypes found solely in Japan. A previously published COI based *D. vexillum* phylogeny [52] is consistent with this conclusion, although as only two clade B haplotypes (H9 and H10) were present in this dataset, the clade structure was not so obvious [52]. The high levels of intraspecific genetic variation that are characteristic of ascidian genomes prevent any confident assessment of whether or not *D. vexillum* clades A and B correspond to cryptic, sibling species or to intra-specific subtypes [60]. A number of recent studies using molecular techniques have concluded that some ascidian species groupings, originally erected on the basis of morphology, actually contain distinct sub-clades [61]–[65]. The levels of COI sequence variation between, and within, *D. vexillum* clades A and B, as estimated by percentage of polymorphic sites, are at the lower end of comparable values reported for other ascidian species (e.g., [62], [63], [66], [67]). Furthermore, the level of *D. vexillum* sequence divergence between COI clades A and B is well below the comparable divergence values for two cryptic species now recognised within the *Ciona intestinalis* grouping [68], [69]. Additionally, clade B *D. vexillum* colonies that were examined in a previous global distribution study were deemed morphologically identical to clade A colonies with respect to all characters studied [49]. Both the COI sequence data and the morphological evidence support an interpretation that *D. vexillum* clades A and B are best regarded, at least for the present, as intra-species variants rather than cryptic species. An apparent lack of inter-clade introgression of two *D. vexillum* 18S rDNA alleles supports clades A and B being distinct species. However, this interpretation is tentative and needs to be supported by looking at additional, independently segregating, nuclear haplotypes from *D. vexillum* clade A and B populations occurring in sympatry in Japan. For the present, the most parsimonious conclusion is that *D. vexillum* COI clades A and B are within the same species grouping and further study is required to resolve this matter.

The lack of an adequate ascidian fossil record prevents calibration of ascidian COI sequence divergence against geological time [69]. However, using the values 1.6–2.6% divergence/million years derived from other marine invertebrate taxa [69] would place divergence of the *D. vexillum* A and B COI clade lineages at 1.5–2.5 million years ago. Furthermore, as several studies have suggested a faster rate of molecular evolution for tunicates than many other organisms, these times may be over-estimates [70]–[73]. It appears likely that *D. vexillum* COI clade A and B lineages diverged within the Pleistocene epoch (2.6 million to 12 000 years BP). Repeated Pleistocene glaciations profoundly affected the oceans around the present day Japanese archipelago [74]. We speculate that clade A and B lineage separation may have occurred in association with such climatic changes – perhaps due to a period of a restricted gene flow between populations in dispersed refugia. It is noteworthy that, from the samples collected to date, *D. vexillum* clade B appears to be restricted to Japan.

While the fusion biology and associated colony allorecognition genetics of the colonial ascidian *B. schlosseri* (fam. Styelidae) has been extensively studied [30], [42] little comparable work has been reported for colonial ascidians from the family Didemnidae. Mukai and Watanabe (1974) [34] found evidence of colony allorecognition specificity in *Didemnum moseleyi* (fam. Didemnidae) whereas Bishop and Sommerfeldt (1999) [38] reported an absence of allorecognition discrimination during colony fusion in *Diplosoma listerianum* (fam. Didemnidae). Rates of chimeric colonies in natural populations of *D. listerianum* were also found to be extremely common [75], [76]. In this study we showed that *D. vexillum* possesses some form of colony recognition mechanism – with autogeneic assays always fusing and some inter-colony fusions resulting in non-fusion reactions. New Zealand populations of *D. vexillum* clade A displayed higher rates of fusion between paired, randomly-selected colonies than was found in Japan. An average of 80% (standard deviation ±18%) of the New Zealand inter-colony CSAs resulted in fusions compared with a value of 27% (standard deviation ±15%) for Japan. Note that in Japan the *D. vexillum* colonies were all from clade A and were collected from within a small bay (i.e. Shizugawa Bay) – a sampling strategy which would bias the sampling of related colonies and thereby inflate the CSA fusion rates. These CSA findings are consistent with a model in which reduction of the allelic diversity at *D. vexillum's* putative allorecognition loci enhances the probability that two randomly selected *D. vexillum* colonies in recently established populations will be genetically similar enough to fuse.

A major challenge facing invasion biology is the description of biological features that may help predict the chances of invasion success and the delineation of the selective advantage(s) such features provide in novel environments [77]. For colonial organisms, an increased rate of inter-colony fusion might be advantageous as such fusions could result in larger, more genetically diverse and, potentially, more adaptable and competitive colonies [41], [78]. Although there may also be evolutionary costs arising from intra-colony somatic cell competition and germ cell parasitism [35]. In this work, we have shown that reduced genetic diversity at a mitochondrial locus in an introduced population of *D. vexillum* is correlated with increased rates of fusion between colonies. Such an enhanced tendency towards forming large chimeric colonies might explain the extreme propensity for biofouling that *D. vexillum* displays outside its putative native range [54], [55]. However, caution is warranted when generalizing the fusions results obtained in this study and it would be illuminating to carry out similar experiments in other invasive populations of *D. vexillum*. In addition, work investigating the frequency of chimeric colonies in both the New Zealand and Japanese *D. vexillum* populations is currently underway. The results of the combined population molecular genetic and phenotype-level study reported here suggest a model by which reductions in genetic diversity may enhance the invasiveness and biofouling propensity of *D. vexillum*. This model also suggests that a potential biocontrol method for *D. vexillum* outside its native range could be the introduction of additional genetic diversity into the invasive populations. Such introductions of additional genetic diversity would not remove *D. vexillum* from non-native regions, an undertaking now widely accepted as impossible, but may attenuate *D. vexillum*'s propensity of biofouling that negatively impacts on aquaculture.

## Materials and Methods

### Ethics statement

No specific permits were required for the described field studies. No locations were privately-owned or protected in any way and the studies did not involve endangered or protected species.

### Tissue sampling for population genetics

Tissue samples were taken from colonies morphologically identified as *D. vexillum* in New Zealand (five locations: Whangamata, Marlborough Sounds, Port Nelson, Wellington Habour, and Lyttelton Harbour) and Japan (four locations: Izu Peninsula, Sagami Bay, Ise Bay, and Shizugawa Bay) between April 2008 and July 2009 (Figure S1, Table S1). Samples were collected from colonies ≥2 m distance apart to minimize the chances of pseudo-replication of sampling from clonally related colonies. Tissue samples (ca. 100–500 mg) were preserved in approx. 2.0 ml of 96% (v/v) ethanol and stored at −20°C.

### Amplification of mitochondrial cytochrome oxidase I and 18S ribosomal gene sequences

Tissue samples (ca. 50 mg) were macerated using a sterile scalpel blade. Total genomic DNA was extracted using i-genomic CTB DNA extraction mini kits (animal tissue protocol; Intron, Gyeonggi-do, South Korea). An approximately 600 bp section of the COI gene was amplified using tunicate primers (Tun_forward and Tun_reverse2) [52]. The polymerase chain reaction (PCR) amplifications were carried out in 50 µl reaction volumes containing; 25 µl of i-Taq 2× PCR master mix (Intron, Gyeonggi-do, Korea), 0.4 µM of both forward and reverse primers and 1.0 µl of template containing ca. 50–150 ng of DNA. Thermocycling conditions consisted of an initial denaturing step of 95°C, 4 min, followed by 40 cycles of 94°C, 1 min; 39°C, 1 min; 72°C, 90 seconds; with a final extension step of 72°C, 10 min. If any COI haplotype sequences were found only once, the samples were re-analysed to ensure the observed differences were not due to sequencing error or replication errors during the PCR. An approximately 900 bp section of the 18S rDNA gene was amplified as two overlapping fragments using the PCR primer pairs F16 with R497 and F476 with R917 [79]. PCR mixes were made up as described above with thermocycling conditions of initial denaturing step of 94°C, 2 minutes, followed by 30 cycles of 94°C, 30 seconds; 56°C, 30 seconds, 72°C, 30 seconds; with a final extension step of 72°C, 10 minutes. Amplification products were purified using AxyPrep PCR cleanup kits (Axygen, California, USA) and sequenced in both directions, using the PCR primers, by an external contractor (Waikato University DNA Sequencing Facility, Hamilton, New Zealand). Sequence chromatograms were examined visually and any base-calling errors corrected manually using the BioEdit Sequence Alignment Editor [80]. Both forward and reverse sequences were aligned and any apparent conflicts resolved by manual inspection. For subsequent analyses the COI and 18S sequence data matrices were truncated to 586 bp and 861 bp respectively.

### Phylogenetic analyses

The initial data for phylogenetic analyses consisted of the 98 new COI sequences generated as part of this study and 71 previously published sequences [52] (GenBank accession numbers EU419401–EU419406, EU419409–EU419431, EU419433–EU419455, EU419457–EU419459, and EU742662–EU742677). Identical sequences were collapsed into single haplotypes to generate a total of 16 haplotypes. COI sequences from *Didemnum psammatode* (EU742661, JN624758), *D. albidum* (EU419432, EU419456) and *Didemnum* sp. B (EU419407, EU419408) were used as outgroups. Bayesian phylogenetic analyses were performed using MrBayes 3.1.2 [81]. The generalized time reversible model with a proportion of invariable sites and a gamma shaped distribution of rates across sites (GTR+I+gamma) was selected using MrModeltest v 2.2 [82]. The Bayesian analysis was carried out in two simultaneous runs for 5×10^6^ generations, with four chains each and trees were sampled every 100 generations. Of the 5×10^4^ trees sampled the latter 4.9×10^3^, were used to construct a 50% majority-rule consensus tree.

Pairwise distances (the proportion of nucleotide sites that differ between two sequences) and average *p*-distances were calculated using MEGA 4.0 [83]. The number of net nucleotide substitutions per site (*D*a) was calculated in DnaSP 5.0 [84].

### Population genetic analyses

Mitochondrial COI sequences were first grouped into five broad geographical areas; New Zealand (NZ), Japan, West Coast North America (WCNA), East Coast North America (ECNA) and Europe. For each geographical region four measures of genetic diversity were calculated using ARLEQUIN 3.01 [85]: (i) total COI haplotype numbers, (ii) numbers of private COI haplotypes, (iii) nucleotide diversity, and (iv) haplotype diversity. Haplotype richness was calculated using FSTAT v2.9.3.2 [86]. Using the program TCS 1.21 [87] a 95% statistical parsimony cladogram network of the 16 haplotypes was built.

### Cut surface assay (CSA) of inter-colony fusion

To assess the rates of inter-colony fusion within populations of *D. vexillum*, cut surface assays (CSA) [43] were performed between colonies collected from New Zealand (Marlborough Sounds) and Japan (Shizugawa Bay) (Figure S1, Table S2). Colonies for fusion assays were collected ≥5 m distance apart to minimize the chances of selecting clonally related colonies. Using a scalpel blade, small pieces (ca. 20 mm×20 mm) were cut from two colonies and attached with cotton thread to a Perspex plate (150 mm×150 mm×5 mm) with the cut edges touching. CSA were set up for all collected colony combinations for each experiment. Plates were hung vertically from floating platforms into seawater in sheltered locations at a depth of 2 m. Colonies were left to grow for 3–4 days and then examined for fusion between the tissue samples from different colonies. Autogeneic assays, using two pieces of tissue from the same colony, were used as positive controls. CSA were recorded as fused if the two paired colony fragments could not be pulled apart after three to four days of growth. Although some CSA pairs initially appeared to fuse, the tissue at the fusion zone subsequently decayed and such transient fusions were recorded as non-fusions. Fusion percentages obtained from CSA experiments within NZ, within Japan and between NZ and Japan were compared using two-sided z-tests. COI sequences from all the colonies used in the CSAs were obtained to confirm their taxonomic identity as *D. vexillum*.

## Supporting Information

Figure S1 ***Didemnum vexillum*** **colony collection sites for DNA sampling and/or cut surface assays (CSA) in New Zealand (A) and Japan (B).** (A) New Zealand sites: Marlborough Sounds (a, Ruakaka Bay; b, Hitaua Bay; c, Onahau Bay; d, Homeward Bay; e, Grant Bay; f, West Beatrix Bay; g, South East Bay; h, Yncyca Bay; I, Fairy Bay; j, Four Fathom Bay; k, Picton; l, Kenepuru Sound); Port Nelson; Wellington Harbour; Port Lyttelton; Whangamata. (B) Japan sites: Shizugawa Bay; Misaki Bay; Izu Peninsula; Ise Bay. Maps are not to scale.
(TIF)

Table S1 **Source and sequence details, including GenBank accession numbers, of the** ***Didemnum vexillum*** **cytochrome oxidase I (COI) sequences from this study.**
(DOC)

Table S2 **Results of cut surface assays (CSA) of** ***Didemnum vexillum*** **colonies from New Zealand (A) and Japan (B).** Collection sites are indicated with the bracketed letters corresponding to locations in Figure S1. Diagonal boxes show autogenic (i.e. positive control) fusions with the colony's COI haplotype, blank boxes below the diagonal indicate inter-colony pairings that resulted in fusion, boxes with a ‘×’ indicate inter-colony pairings that did not result in fusion.
(DOC)

## References

[1] Mooney HA, Cleland EE (2001). The evolutionary impact of invasive species.. Proc Natl Acad Sci U S A.

[2] Pimentel D, Lach L, Zuniga R, Morrison D (2000). Environmental and economic costs of nonindigenous species in the United States.. Bioscience.

[3] Vitousek PM, Mooney HA, Lubchenco J, Melillo JM (1997). Human domination of Earth's ecosystems.. Science.

[4] Sakai AK, Allendorf FW, Holt JS, Lodge DM, Molofsky J (2001). The population biology of invasive species.. Annu Rev Ecol Syst.

[5] Nentwig W (2007). Biological Invasions. Ecological Studies 193.

[6] Estoup A, Guillemaud T (2010). Reconstructing routes of invasion using genetic data: why, how and so what?. Mol Ecol.

[7] Nei M, Maruyama T, Chakraborty R (1975). The bottleneck effect and genetic variability in populations.. Evolution.

[8] Dlugosch KM, Parker IM (2008). Founding events in species invasions: genetic variation, adaptive evolution, and the role of multiple introductions.. Mol Ecol.

[9] Roman J, Darling JA (2007). Paradox lost: genetic diversity and the success of aquatic invasions.. Trends Ecol Evol.

[10] Lee CE (2002). Evolutionary genetics of invasive species.. Trends Ecol Evol.

[11] Geller JB, Darling JA, Carlton JT (2010). Genetic perspectives on marine biological invasions.. Ann Rev Mar Sci.

[12] Crawford KM, Whitney KD (2010). Population genetic diversity influences colonization success.. Mol Ecol.

[13] Sax DF, Brown JH (2000). The paradox of invasion.. Global Ecol Biogeogr.

[14] Puillandre N, Dupas S, Dangles O, Zeddam JL, Capdevielle-Dulac C (2008). Genetic bottleneck in invasive species: the potato tuber moth adds to the list.. Biol Invasions.

[15] Zhan AB, Macisaac HJ, Cristescu ME (2010). Invasion genetics of the *Ciona intestinalis* species complex: from regional endemism to global homogeneity.. Mol Ecol.

[16] Kolbe JJ, Glor RE, Schettino LRG, Lara AC, Larson A (2004). Genetic variation increases during biological invasion by a Cuban lizard.. Nature.

[17] Kelly DW, Muirhead JR, Heath DD, MacIsaac HJ (2006). Contrasting patterns in genetic diversity following multiple invasions of fresh and brackish waters.. Mol Ecol.

[18] Voisin M, Engel CR, Viard F (2005). Differential shuffling of native genetic diversity across introduced regions in a brown alga: aquaculture vs. maritime traffic effects.. Proc Natl Acad Sci U S A.

[19] Colautti RI, Ricciardi A, Grigorovich IA, Macisaac HJ (2004). Is invasion success explained by the enemy release hypothesis?. Ecol Lett.

[20] Blossey B, Notzold R (1995). Evolution of increased competitive ability in invasive nonindigenous plants: a hypothesis.. J Ecol.

[21] Salo P, Korpimaki E, Banks PB, Nordstrom M, Dickman CR (2007). Alien predators are more dangerous than native predators to prey populations.. P Roy Soc B - Biol Sci.

[22] Cote J, Fogarty S, Weinersmith K, Brodin T, Sih A (2010). Personality traits and dispersal tendency in the invasive mosquitofish (*Gambusia affinis*).. P Roy Soc B - Biol Sci.

[23] Forslund H, Wikstrom SA, Pavia H (2010). Higher resistance to herbivory in introduced compared to native populations of a seaweed.. Oecologia.

[24] Phillips BL, Brown GP, Shine R (2010). Evolutionarily accelerated invasions: the rate of dispersal evolves upwards during the range advance of cane toads.. J Evolution Biol.

[25] Van Bocxlaer I, Loader SP, Roelants K, Biju SD, Menegon M (2010). Gradual adaptation toward a range-expansion phenotype initiated the global radiation of toads.. Science.

[26] van Wilgenburg E, Torres CW, Tsutsui ND (2010). The global expansion of a single ant supercolony.. Evol Appl.

[27] Winkler G, Dodson JJ, Lee CE (2008). Heterogeneity within the native range: population genetic analyses of sympatric invasive and noninvasive clades of the freshwater invading copepod *Eurytemora affinis*.. Mol Ecol.

[28] Gotzek D, Ross KG (2007). Genetic regulation of colony social organization in fire ants: an integrative overview.. Q Rev Biol.

[29] Benabentos R, Hirose S, Sucgang R, Curk T, Katoh M (2009). Polymorphic members of the lag gene family mediate kin discrimination in *Dictyostelium*.. Curr Biol.

[30] McKitrick TR, De Tomaso AW (2010). Molecular mechanisms of allorecognition in a basal chordate.. Semin Immunol.

[31] Tsutsui ND, Suarez AV, Holway DA, Case TJ (2000). Reduced genetic variation and the success of an invasive species.. Proc Natl Acad Sci U S A.

[32] Helanterä H, Strassmann JE, Carrillo J, Queller DC (2009). Unicolonial ants: where do they come from, what are they and where are they going?. Trends Ecol Evol.

[33] Corin SE, Abbott KL, Ritchie PA, Lester PJ (2007). Large scale unicoloniality: the population and colony structure of the invasive Argentine ant (*Linepithema humile*) in New Zealand.. Insect Soc.

[34] Mukai H, Watanabe H (1974). On the occurrence of colony specificity in some compound ascidians.. Biol Bull.

[35] Grosberg RK (1988). The evolution of allorecognition specificity in clonal invertebrates.. Q Rev Biol.

[36] Saito Y, Hirose E, Watanabe H (1994). Allorecognition in compound ascidians.. Int J Dev Biol.

[37] Stoner DS, Weissman IL (1996). Somatic and germ cell parasitism in a colonial ascidian: possible role for a highly polymorphic allorecognition system.. Proc Natl Acad Sci U S A.

[38] Rinkevich B (2005). Natural chimerism in colonial urochordates.. J Exp Mar Biol Ecol.

[39] Ben-Shlomo R (2008). The molecular basis of allorecognition in ascidians.. Bioessays.

[40] Bishop JDD, Sommerfeldt AD (1999). Not like *Botryllus*: indiscriminate post-metamorphic fusion in a compound ascidian.. P Roy Soc Lond B Bio.

[41] Rinkevich B, Yankelevich I (2004). Environmental split between germ cell parasitism and somatic cell synergism in chimeras of a colonial urochordate.. J Exp Biol.

[42] De Tomaso AW, Nyholm SV, Palmeri KJ, Ishizuka KJ, Ludington WB (2005). Isolation and characterization of a protochordate histocompatibility locus.. Nature.

[43] Rinkevich B (2005). Rejection patterns in botryllid ascidian immunity: the first tier of allorecognition.. Can J Zool.

[44] Payne CM, Tillberg CV, Suarez AV (2004). Recognition systems and biological invasions.. Ann Zool Fenn.

[45] Ben-Shlomo R, Motro U, Paz G, Rinkevich B (2008). Pattern of settlement and natural chimerism in the colonial urochordate *Botryllus schlosseri*.. Genetica.

[46] Coffey BT (2001). Potentially invasive compound ascidian, Whangamata Harbour.

[47] Kott P (2002). A complex didemnid ascidian from Whangamata, New Zealand.. J Mar Biol Assoc UK.

[48] Gittenberger A (2007). Recent population expansions of non-native ascidians in The Netherlands.. J Exp Mar Biol Ecol.

[49] Lambert G (2009). Adventures of a sea squirt sleuth: unraveling the identity of *Didemnum vexillum*, a global ascidian invader.. Aquat Invasions.

[50] Minchin D, Sides E (2006). Appearance of a cryptic tunicate, a *Didemnum* sp. fouling marina pontoons and leisure craft in Ireland.. Aquat Invasions.

[51] Bullard SG, Lambert G, Carman MR, Byrnes J, Whitlatch RB (2007). The colonial ascidian *Didemnum* sp. A: current distribution, basic biology and potential threat to marine communities of the northeast and west coasts of North America.. J Exp Mar Biol Ecol.

[52] Stefaniak L, Lambert G, Gittenberger A, Zhang H, Lin S (2009). Genetic conspecificity of the worldwide populations of *Didemnum vexillum* Kott, 2002.. Aquat Invasions.

[53] Coutts ADM, Forrest BM (2007). Development and application of tools for incursion response: lessons learned from the management of the fouling pest *Didemnum vexillum*.. J Exp Mar Biol Ecol.

[54] Valentine PC, Carman MR, Blackwood DS, Heffron EJ (2007). Ecological observations on the colonial ascidian *Didemnum* sp. in a New England tide pool habitat.. J Exp Mar Biol Ecol.

[55] Coutts ADM (2002). A biosecurity investigation of a barge in the Marlborough Sounds.

[56] Chapman JW, Carlton JT (1991). A test of criteria for introduced species: the global invasion by the isopod *Synidotea laevidorsalis* (Miers, 1881).. J Crustacean Biol.

[57] Silva N, Smith WC (2008). Inverse correlation of population similarity and introduction date for invasive ascidians.. PLoS ONE.

[58] Darling JA, Bagley MJ, Roman J, Tepolt CK, Geller JB (2008). Genetic patterns across multiple introductions of the globally invasive crab genus *Carcinus*.. Mol Ecol.

[59] Ramstad KM, Woody CA, Habicht C, Sage GK, Seeb JE (2007). Concordance of nuclear and mitochondrial DNA markers in detecting a founder event in Lake Clark sockeye salmon.. Am Fish S S.

[60] Pérez-Portela R, Turon X (2008). Cryptic divergence and strong population structure in the colonial invertebrate *Pycnoclavella communis* (Ascidiacea) inferred from molecular data.. Zoology.

[61] Tarjuelo I, Posada D, Crandall KA, Pascual M, Turon X (2001). Cryptic species of *Clavelina* (Ascidiacea) in two different habitats: harbours and rocky littoral zones in the northwestern Mediterranean.. Mar Biol.

[62] Tarjuelo I, Posada D, Crandall KA, Pascual M, Turon X (2004). Phylogeography and speciation of colour morphs in the colonial ascidian *Pseudodistoma crucigaster*.. Mol Ecol.

[63] López-Legentil S, Turon X (2005). How do morphotypes and chemotypes relate to genotypes? The colonial ascidian *Cystodytes* (Polycitoridae).. Zool Scr.

[64] Caputi L, Andreakis N, Mastrototaro F, Cirino P, Vassillo M (2007). Cryptic speciation in a model invertebrate chordate.. Proc Natl Acad Sci U S A.

[65] Iannelli F, Griggio F, Pesole G, Gissi C (2007). The mitochondrial genome of *Phallusia mammillata* and *Phallusia fumigata* (Tunicata, Ascidiacea): high genome plasticity at intra-genus level.. BMC Evol Biol.

[66] López-Legentil S, Turon X, Planes S (2006). Genetic structure of the star sea squirt, *Botryllus schlosseri*, introduced in southern European harbours.. Mol Ecol.

[67] Turon X, Tarjuelo I, Duran S, Pascual M (2003). Characterising invasion processes with genetic data: an Atlantic clade of *Clavelina lepadiformis* (Ascidiacea) introduced into Mediterranean harbours.. Hydrobiologia.

[68] Nydam ML, Harrison RG (2010). Polymorphism and divergence within the ascidian genus *Ciona*.. Mol Phylogenet Evol.

[69] Nydam ML, Harrison RG (2010). Introgression despite substantial divergence in a broadcast spawning marine invertebrate.. Evolution.

[70] Winchell CJ, Sullivan J, Cameron CB, Swalla BJ, Mallatt J (2002). Evaluating hypotheses of deuterostome phylogeny and chordate evolution with new LSU and SSU ribosomal DNA data.. Mol Biol Evol.

[71] Yokobori S, Oshima T, Wada H (2005). Complete nucleotide sequence of the mitochondrial genome of *Doliolum nationalis* with implications for evolution of urochordates.. Mol Phylogenet Evol.

[72] Delsuc F, Brinkmann H, Chourrout D, Philippe H (2006). Tunicates and not cephalochordates are the closest living relatives of vertebrates.. Nature.

[73] Tsagkogeorga G, Turon X, Galtier N, Douzery EJP, Delsuc F (2010). Accelerated evolutionary rate of housekeeping genes in tunicates.. J Mol Evol.

[74] Nakagawa H (1965). Pleistocene sea levels along the Pacific coast of Japan.. Sci Rep Tohoku Univ.

[75] Sommerfeldt AD, Bishop JDD (1999). Random amplified polymorphic DNA (RAPD) analysis reveals extensive natural chimerism in a marine protochordate.. Mol Ecol.

[76] Sommerfeldt AD, Bishop JDD, Wood CA (2003). Chimerism following fusion in a colonial ascidian (Urochordata).. Biol J Linn Soc.

[77] Williamson MH, Fitter A (1996). The characters of successful invaders.. Biol Conserv.

[78] Westerman EL, Dijkstra JA, Harris LG (2009). High natural fusion rates in a botryllid ascidian.. Mar Biol.

[79] Price A, Collie JS, Smith DC (2005). 18S ribosomal RNA and cytochrome oxidase gene sequences of *Didemnum* sp., an invasive colonial tunicate. GSO Technical Report No. 2006-01, Summer Undergraduate Research Fellowship Program in Oceanography.

[80] Hall TA (1999). BioEdit: a user-friendly biological sequence alignment editor and analysis program for Windows 95/98/NT.. Nucl Acid S.

[81] Huelsenbeck JP, Ronquist F (2001). MRBAYES: Bayesian inference of phylogenetic trees.. Bioinformatics.

[82] Nylander JAA (2004). MrModeltest v2 program distributed by the author. Evolutionary Biology Centre.

[83] Tamura K, Dudley J, Nei M, Kumar S (2007). Molecular evolutionary genetics analysis (MEGA) software version 4.0.. Mol Biol Evol.

[84] Rozas J, Sánchez-Del Barrio JC, Messeguer X, Rozas R (2003). DnaSP, DNA polymorphism analyses by the coalescent and other methods.. Bioinformatics.

[85] Excoffier L, Laval G, Schneider S (2005). Arlequin (version 3.0): an integrated software package for population genetics data analysis.. Evol Bioinform.

[86] Goudet J (1995). FSTAT (Version 1.2): A computer program to calculate F-statistics.. J Hered.

[87] Clement M, Posada D, Crandall KA (2000). TCS: a computer program to estimate gene genealogies.. Mol Ecol.

