# Supplementary material for: Increased Inter-Colony Fusion Rates Are Associated with Reduced COI Haplotype Diversity in an Invasive Colonial Ascidian Didemnum vexillum
Source: PLoS One. 2012 Jan 31;7(1):e30473. doi: 10.1371/journal.pone.0030473 (PMC3269411; doi:10.1371/journal.pone.0030473)
Supplement: Table S1 — Source and sequence details, including GenBank accession numbers, of the Didemnum vexillum cytochrome oxidase I (COI) sequences from this study. (DOC) [file pone.0030473.s002.doc]

| **Haplotype** | **Accession number** | **Location** | **Approx. coordinates** | **Date** | **No. of samples** |
| --- | --- | --- | --- | --- | --- |
| 2 | JF738057 | Shizugawa Bay, Japan | 38°38.4'N, 141°27.2'E | 07/07/09 | 6 |
| 3 | JF738058 | Ise Bay, Japan | 34°29.0'N, 136°52.3'E | 30/06/09 | 3 |
|  |  | Shizugawa Bay, Japan | 38°38.4'N, 141°27.2'E | 07/07/09 | 2 |
|  |  | Marlborough Sounds, NZ | 41°08.4'S, 173°59.11'E | 04/08 to 04/09 | 29 |
|  |  | Wellington, NZ | 41°17.0'S, 174°46.4'E | 08/03/09 | 1 |
|  |  | Nelson, NZ | 41°15.2'S, 173°16.3'E | 25/03/09 | 1 |
|  |  | Whangamata Habour, NZ | 37°12.0'S, 175°52.4'E | 08/12/08 | 14 |
|  |  | Lyttelton, NZ | 43°36.2'S, 172°43.0'E | 01/06/08 | 1 |
| 5 | JF738059 | Shizugawa Bay, Japan | 38°38.4'N, 141°27.2'E | 06/07/09 | 6 |
|  |  | Marlborough Sounds, NZ | 41°08.4'S, 173°59.11'E | 04/08 to 04/09 | 15 |
| 6 | JF738060 | Shizugawa Bay, Japan | 38°38.4'N, 141°27.2'E | 07/07/09 | 1 |
| 9 | JF738061 | Sagami Bay, Japan | 35°09.3'N, 139°36.4'W | 12/06/09 | 5 |
| 10 | JF738062 | Sagami Bay, Japan | 35°09.3'N, 139°36.4'W | 12/06/09 | 1 |
| 11 | JF738063 | Izu Peninsula, Japan | 34°39.6'N, 138°56.2'W | 25/05/09 | 3 |
| 12 | JF738064 | Izu Peninsula, Japan | 34°39.6'N, 138°56.2'W | 12/06/09 | 2 |
| 13 | JF738065 | Ise Bay, Japan | 34°29.0'N, 136°52.3'E | 30/06/09 | 1 |
| 14 | JF738066 | Sagami Bay, Japan | 35°09.5'N, 139°36.7'W | 15/06/09 | 1 |
|  |  | Izu Peninsula, Japan | 34°39.6'N, 138°56.2'W | 19/06/09 | 3 |
| 15 | JF738067 | Izu Peninsula, Japan | 34°39.6'N, 138°56.2'W | 19/06/09 | 1 |
| 16 | JF738068 | Shizugawa Bay, Japan | 38°38.4'N, 141°27.2'E | 07/07/09 | 1 |
| 17 | JF738069 | Ise Bay, Japan | 34°29.0'N, 136°52.3'E | 30/07/09 | 1 |
|  |  |  |  | **Total** | 98 |
