# Supplementary material for: Increased Inter-Colony Fusion Rates Are Associated with Reduced COI Haplotype Diversity in an Invasive Colonial Ascidian Didemnum vexillum
Source: PLoS One. 2012 Jan 31;7(1):e30473. doi: 10.1371/journal.pone.0030473 (PMC3269411; doi:10.1371/journal.pone.0030473)
Supplement: Table S2 — Results of cut surface assays (CSA) of Didemnum vexillum colonies from New Zealand (A) and Japan (B). Collection sites are indicated with the bracketed letters corresponding to locations in Figure S1. Diagonal boxes show autogenic (i.e. positive control) fusions with the colony's COI haplotype, blank boxes below the diagonal indicate inter-colony pairings that resulted in fusion, boxes with a ‘×’ indicate inter-colony pairings that did not result in fusion. (DOC) [file pone.0030473.s003.doc]

A. New Zealand

| 27 Feb 2009 | Ruakaka 1 (a) | Ruakaka 2 (a) | Hitaua 1 (b) | Hitaua 2 (b) | Onahau (c) |
| --- | --- | --- | --- | --- | --- |
| Ruakaka 1 (a) | H3 |  |  |  |  |
| Ruakaka 2 (a) |  | H3 |  |  |  |
| Hitaua 1 (b) |  |  | H3 |  |  |
| Hitaua 1 (b) | × |  |  | H3 |  |
| Onahau (c) | × |  | × |  | H3 |

| 6 Mar 2009 | Homeward (d) | Grant (e) | W Beatrix (f) | South East (g) | Yncyca 1 (h) | Fairy (i) | Fathom (j) |
| --- | --- | --- | --- | --- | --- | --- | --- |
| Homeward (d) | H3 |  |  |  |  |  |  |
| Grant (e) | × | H3 |  |  |  |  |  |
| W Beatrix (f) |  |  | H5 |  |  |  |  |
| South East (g) | × |  |  | H3 |  |  |  |
| Yncyca 1 (h) |  |  |  |  | H5 |  |  |
| Fairy (i) |  |  |  |  |  | H3 |  |
| Fathom (j) |  |  |  |  |  |  | H5 |

| 25 Mar 2009 | Ruakaka 3 (a) | Ruakaka 4 (a) | Nelson | Picton 1 (k) | Picton 2 (k) |
| --- | --- | --- | --- | --- | --- |
| Ruakaka 3 (a) | H3 |  |  |  |  |
| Ruakaka 4 (a) |  | H5 |  |  |  |
| Nelson |  | × | H3 |  |  |
| Picton 1 (k) | × | × |  | H5 |  |
| Picton 2 (k) | × |  |  |  | H5 |

| 6 Apr 2009 | Kenepuru Sound (l) | Yncyca 2 (h) | Yncyca 3 (h) |
| --- | --- | --- | --- |
| Kenepuru Sound (l) | H3 |  |  |
| Yncyca 2 (h) |  | H3 |  |
| Yncyca 3 (h) |  |  | H5 |

B. Japan

| 9 Jul 2010 | Shizugawa 1 | Shizugawa 2 | Shizugawa 3 | Shizugawa 4 | Shizugawa 5 |
| --- | --- | --- | --- | --- | --- |
| Shizugawa 1 | H2 |  |  |  |  |
| Shizugawa 2 |  | H2 |  |  |  |
| Shizugawa 3 |  | × | H2 |  |  |
| Shizugawa 4 | × | × | × | H5 |  |
| Shizugawa 5 |  | × |  | × | H5 |

| 12 Jul 2010 | Shizugawa 6 | Shizugawa 7 | Shizugawa 8 | Shizugawa 9 | Shizugawa 10 |
| --- | --- | --- | --- | --- | --- |
| Shizugawa 6 | H5 |  |  |  |  |
| Shizugawa 7 | × | H5 |  |  |  |
| Shizugawa 8 | × | × | H5 |  |  |
| Shizugawa 9 | × | × | × | H5 |  |
| Shizugawa 10 | × | × | × |  | H5 |

| 13 Jul 2010 | Shizugawa 11 | Shizugawa 12 | Shizugawa 13 | Shizugawa 14 | Shizugawa 15 |
| --- | --- | --- | --- | --- | --- |
| Shizugawa 11 | H5 |  |  |  |  |
| Shizugawa 12 | × | H5 |  |  |  |
| Shizugawa 13 | × | × | H5 |  |  |
| Shizugawa 14 |  | × | × | H5 |  |
| Shizugawa 15 |  | × |  | × | H5 |
